# Multigroup Comparisons with Configural Frequency Analysis

**DOI:** 10.17505/jpor.2025.27572

**Published:** 2025-04-01

**Authors:** Alexander von Eye, Wolfgang Wiedermann

**Affiliations:** 1 Michigan State University, USA; 2 University of Missouri, Columbia, USA

**Keywords:** Configural Frequency Analysis, CFA, two-group CFA, multiple group CFA, CFA base models

## Abstract

Lienert’s (1973) original approach to comparing groups with Configural Frequency Analysis (CFA) cannot straightforwardly be generalized to the comparison of multiple groups. The present article proposes a new base model for group comparison with CFA. This model allows researchers to compare multiple groups, to evaluate overall model fit, to take covariates into account, and to conduct exploratory and confirmatory analyses. In confirmatory group comparisons, base models need to be specified in which particular configurations are blanked out, and other configurations are explicitly set equal. Reference is made to existing base models, e.g., the configural model of axial symmetry. Data examples are provided in which individuals are compared. Extensions of the new models are discussed.

Configural Frequency Analysis (CFA; Lienert, 1968; Lienert & Krauth, 1975; von Eye & Gutiérrez Peña, 2004; von Eye et al., 2010; von Eye & Wiedermann, 2021), mostly applied in person-oriented research (Bergman et al., 2003; von Eye et al., 2015; von Eye & Wiedermann, 2025), allows researchers to test hypotheses concerning the density in sectors of the data space. These sectors are termed *configurations*, and they are defined by patterns of variable categories. When the density in a particular configuration is higher than hypothesized, CFA has identified a *type*. When the density is lower than hypothesized, CFA has identified an *antitype*. The search for types and antitypes can be performed in both exploratory and confirmatory research.

CFA hypotheses are based on a particular probability model, the *CFA base model*. Although, over the years, many base models have been specified, the first version of a general CFA base model has been formulated only recently (von Eye & Wiedermann, 2025). A CFA base model contains all effects that a researcher is *not* interested in. When the model is rejected, effects that the researcher is interested in are bound to exist, statistically.

There exists a small number of approaches to CFA for which no base model has been proposed that would lead directly to the identification of types and antitypes. Most prominent among these is two-group CFA (Lienert, 1973, 1985; von Eye et al., 1995). Application of this approach only involves performing tests to compare individual configurations. These tests can be selected from a number of options that are unrelated to two-group CFA itself. That is, they can be and have been applied in other contexts than CFA as well.

In this article, we first propose a new approach to two-group CFA. In this approach, the comparison of configurations is part of a particular base model. Second, we propose extending two-group CFA to accommodate multiple groups. For the extended model, a base model is proposed as well. This article is structured as follows. First, we briefly review Lienert’s (1973) original two-group CFA. Then, we present the new base models, that is, the base models for two- and multiple group CFA. For each approach, real-world data examples are given.

## Lienert’s (1973) Original Two-Group CFA

To compare individual configurations in two groups of responses or respondents, Lienert’s (1973) original approach sets up group by configuration cross-classifications. That is, when *t* variables are crossed with two groups, one obtains a cross-table with *c* = *c*_1_ × *c*_2_ × … × *c_t_* × 2 cells, where *c_t_* is the number of categories of the *i*th variable. Two-group CFA involves the application of tests to compare the two groups in each of the *c_T_* = *c*_1_ × *c*_2_ × … × *c_t_* configurations. When a configuration suggests discrepancies, that is, differences from the proportions suggested by the sizes of the groups, it is said to constitute a *discrimination type*. In this version of group comparison by CFA, there are no *discrimination antitypes* because when one group contains disproportionately many cases, the other group must contain disproportionately few cases.

To determine whether discrimination types exist, a number of tests has been proposed. These include Fisher’s exact test, variants of the Pearson Chi-square test, the *z*-test, the binomial test, the odds ratio, and three versions of the log-linear *λ* (Goodman, 1991). A comparison of these tests was presented by von Eye et al. (1995). This comparison also included correlation coefficients. Results of this comparison suggest that, in the context of two-group CFA, Fisher’s exact test and the *z*-test may be the most powerful ones. To select from these tests, however, it must be considered, in addition to power, that, whereas the odds ratio and one of the *λ* measures are marginal-free, the other tests and the correlations are marginal-dependent. Marginal-dependent tests (e.g., the Chi-square test) do take the marginal proportions into account, whereas marginal-free tests (e.g., the odds ratio) do not.

In all applications of CFA that involve more than one significance test, the nominal significance threshold *α* is protected based on the number of tests performed. In exploratory two-group CFA, this number is given by the number of configurations in the cross-classification of the *t* variables, that is, *c_T_* = *c*1 × *c*2 × … × *c_t_*. In a standard CFA of the same table, the number of tests would be *c* = *c_T_* × 2. Evidently, in two-group CFA, there is only half the number of tests and, therefore, a less strict adjusted significance threshold than in standard CFA. The adjusted threshold can be determined using, for example, the Bonferroni (1936) or the Holland Di Ponzio-Copenhaver (1987) procedures. In confirmatory CFA, the number of tests can be even smaller. The protected significance threshold will, therefore, be even less extreme.

## Data Example

In the following examples, we use data that were collected in a longitudinal study on the development of alcohol consumption (Perrine, et al., 1995). A sample of male respondents who had identified themselves as alcoholics indicated, on a daily basis, the amount of alcohol they had consumed the day before. Here, we take a person-oriented perspective and compare the two respondents labeled 3000 and 3004. We ask whether the relation of beer consumption and stress is the same for the two respondents. Originally, beer consumption was coded in number of 0.33 l-size beers consumed the day before the automated phone interview, and stress was coded on a 10-point scale with 10 indicating maximal stress. To obtain a smaller-size cross-classification and to avoid large numbers of empty cells, beer consumption was recoded into the three categories 1 = three beers consumed or less, 2 = between four and seven beers consumed, and 3 = more than seven beers consumed. Stress was recoded into the three categories 1 = no to moderate level stress (less than original level five), 2 = moderate level stress (between five and seven), and 3 = high stress (original levels eight and up). For the analyses we use the answers respondent 3000 has provided on 735 consecutive days and those that respondent 3004 has provided on 742 consecutive days. For the two-group CFA as proposed by Lienert (1973), we use the *z*-test and protect *α* using the procedure proposed by Holland and Di Ponzio Copenhaver (1987). Table 1 displays the results of this analysis.

**Table 1:** Two-group CFA for the Comparison of Respondents 3000 and 3004 in the Relation between Beer Consumption and Stress (B = Beer Consumption, S = Stress, G = Group)

Configuration				
BSG	*m*	*z*	*p*	Type?
111	263,00			Discrimination
112	3.00	17.691	.000000	Type

121	408.00			Discrimination
122	124.00	15.530	.000000	Type

131	19.00			Discrimination
132	109.00	-8.268	.000000	Type

211	21.00			Discrimination
212	4.00	3.453	.000277	Type

221	23.00			Discrimination
222	182.00	-11.894	.000000	Type

231	.00			Discrimination
232	123.00	-11.529	.000000	Type

311	1.00			
312	7.00	-2.114	.017272		
321	.00			Discrimination
322	121.00	-11.426	.000000	Type

331	.00			Discrimination
332	69.00	-8.467	.000000	Type


In Table 1, the comparison pairs are placed in subsequent lines. The table suggests that the two respondents differ in all but one pattern of beer consumption and stress. In the first comparison pair, we see the most extreme difference between the two individuals, with *m*_111_ = 263 days for Respondent 3000 and *m*_112_ = 3 days for respondent 3004. The *z*-score for the comparison of these two frequencies is *z* = 17.691 and the corresponding probability is *p* < 0.001. This result suggests that Respondent 3000 reported significantly more days than Respondent 3004 on which he experienced low stress and consumed comparatively small numbers of beers. The configuration with the second highest *z*-score is the one with variable categories 1 2. These are days with small numbers of beers consumed and mid-range stress (*z* = 15.530; *p* < 0.001). Respondent 3000 reports 408 days with this pattern, Respondent 3004 reports 124 days.

Thus far, two-group CFA is the only method available for the comparison of two data sources (groups or individuals) in their response patterns. The comparison can be uni- or multivariate. When more than one source of data is examined, $\left(\begin{array}{c} G\\ 2 \end{array}\right)$, two-group comparisons can be conducted,

where *G* indicates the number of groups. The downside of this approach is that the number of tests involved increases exponentially, and the adjusted significance threshold becomes extreme. In addition, performing multiple two-group comparisons does not answer the same questions as performing a multi-group comparison. Therefore, we propose, in this article, an approach to two- and multigroup CFA that does allow one to compare two and more than two groups.

## A Base Model for Two-group CFA

As is the characteristic of CFA, the approach proposed here involves a series of steps. In the first, a base model is specified. This serves the estimation of expected cell frequencies. In a second step, tests are performed to compare two groups of cases in particular configurations. In the new approach, a log-linear CFA base model must be specified that differs from the base models specified in CFA thus far. Specifically, the new base model represents the null hypotheses for the comparison of corresponding configurations. This hypothesis posits that there is no difference. In exploratory two-group CFA, all comparisons are included. In confirmatory CFA, only a priori selected pairs of configurations are included. The new base model has the following characteristics:

1. the model is saturated in the *t* variables that are used for the comparison of the two groups (these are termed *discrimination variables*);
2. the model is also saturated in the variables that constitute the comparison groups (when there is only one variable that constitutes the groups, the main effects of this variable are included in the base model, when needed); and
3. the model does not include standard interaction terms that would link the discrimination variables and the two groups;
4. instead, the model includes vectors that represent the null hypotheses for the comparisons of corresponding configurations; in exploratory two-group CFA, only the effect listed in (1) need to be included in the base model.

When this base model is rejected, the two groups are bound to differ in particular configurations. When a configuration stands out so that its observed frequencies differ significantly from expectation, it is said to constitute a CFA *discrimination type*. As in Lienert’s (1973) original two-group CFA, there is no distinction between discrimination types and antitypes. The number of tests performed is also the same, and so is the protected significance threshold.

The vectors that represent the comparisons of corresponding configurations are specified such that they represent the null hypothesis of a comparison. This hypothesis posits that there is no difference. According to this hypothesis, the model will result in expected frequencies that are equal for the corresponding configurations. Discrimination types result when these expected frequencies differ significantly from the observed ones. As the new approach to two-group CFA can also result only in discrimination *types*, the distinction between discrimination types and antitypes becomes relevant when three or more groups are compared.

### Data Example

To illustrate the new approach, we re-analyze the data in Table 1. We ask the same question as in the first analysis, but we employ the method just outlined. To specify the base model, we set up a design matrix with the following properties:

1. it contains the main effects of the two variables used to distinguish between Respondents 2000 and 3004, that is, Beer Consumption and Stress; this is needed to prevent the analysis from showing discrimination types that are due to characteristics of the marginal distributions;
2. it contains the interaction between Beer consumption and Stress; this is needed because discrimination types must not emerge just because this interaction exists (the Pearson correlation between Beer Consumption and Stress is *r* = 0.279);
3. it contains vectors that set equal corresponding configurations; discrimination types can, then, emerge when corresponding configurations are unequal; technically, to set equal corresponding configurations, a vector is included in the design matrix that contains the same value for each pairling and zeros else (see vectors 1 2 through 3 2 in Table 2, below); when one configuration is observed more frequently than expected, the corresponding other one will have been observed less frequently; therefore, again, there can only be discrimination types, not discrimination antitypes;
4. it contains vectors that blank out diagonal cells (or any other cells that researchers wish to remove from the two-group comparison).

**Table 2:** Elements of the design matrix for a 3 × 3 × 2 two-group CFA

Main Effects Beer and Stress				Interaction Beer by Stress				Main effect Respondents	Setting equal corresponding configurations						Blanking out configurations		
B1	B2	S1	S2	B1 × S1	B1 × S2	B2 × S1	B2 × S2	G	12	13	21	23	31	32	11	22	33
1	0	1	0	1	0	0	0	1	0	0	0	0	0	0	1	0	0
1	0	1	0	1	0	0	0	-1	0	0	0	0	0	0	0	0	0
1	0	1	0	1	0	0	0	1	1	0	0	0	0	0	0	0	0
1	0	0	1	0	1	0	0	-1	1	0	0	0	0	0	0	0	0
1	0	0	1	0	1	0	0	1	0	1	0	0	0	0	0	0	0
1	0	0	1	0	1	0	0	-1	0	1	0	0	0	0	0	0	0
0	1	-1	-1	0	0	-1	-1	1	0	0	1	0	0	0	0	0	0
0	1	-1	-1	0	0	-1	-1	-1	0	0	1	0	0	0	0	0	0
0	1	-1	-1	0	0	-1	-1	1	0	0	0	0	0	0	0	1	0
0	1	1	0	0	0	1	0	-1	0	0	0	0	0	0	0	0	0
0	1	1	0	0	0	1	0	1	0	0	0	1	0	0	0	0	0
0	1	1	0	0	0	1	0	-1	0	0	0	1	0	0	0	0	0
-1	-1	0	1	0	-1	0	-1	1	0	0	0	0	1	0	0	0	0
-1	-1	0	1	0	-1	0	-1	-1	0	0	0	0	1	0	0	0	0
-1	-1	0	1	0	-1	0	-1	1	0	0	0	0	0	1	0	0	0
-1	-1	-1	-1	1	1	1	1	-1	0	0	0	0	0	1	0	0	0
-1	-1	-1	-1	1	1	1	1	1	0	0	0	0	0	0	0	0	1
-1	-1	-1	-1	1	1	1	1	-1	0	0	0	0	0	0	0	0	0

The last specification, that is, the option of blanking out cells, is a characteristic of *confirmatory* 2-group CFA. Diagonal cells can certainly be included in a group comparison. In the present example, there is no substantive reason to blank out the diagonal cells. It is done solely to illustrate that the resulting base model is comparable to the one discussed by Levendosky and colleagues (2011). These authors discussed a model of axial symmetry for the analysis of repeated observations of attachment of children and adolescents. In this model, those off-diagonal cells are compared that represent the same variable category, observed at different points in time. In the third example, below, we extend this approach to three or more groups of respondents and, thus, to three or more observation points in time. In addition to confirmatory group comparisons (Table 4), we also illustrate exploratory multigroup comparisons (Table 5).

**Table 4:** Three-group CFA for the comparison of three respondents in their Beer consumption and Stress-related responses

*B S G*	*m*	$\hat{m}$	*z*
1 1 1	263.000	263.000	0.000
1 1 2	3.000	3.000	-0.000
1 1 3	72.000	72.000	0.000
1 2 1	408.000	229.667	11.767
1 2 2	124.000	229.667	-6.973
1 2 3	157.000	229.667	-4.795
1 3 1	19.000	46.333	-4.016
1 3 2	117.000	46.333	10.382
1 3 3	3.000	46.333	-6.366
2 1 1	21.000	44.667	-3.541
2 1 2	4.000	44.667	-6.085
2 1 3	109.000	44.667	9.626
2 2 1	23.000	23.013	-0.003
2 2 2	182.000	181.990	0.001
2 2 3	57.000	56.997	0.000
2 3 1	0.000	0.000	-0.000
2 3 2	123.000	65.000	7.194
2 3 3	7.000	65.000	-7.194
3 1 1	1.000	95.000	-9.644
3 1 2	7.000	95.000	-9.029
3 1 3	277.000	95.000	18.673
3 2 1	0.000	55.667	-7.461
3 2 2	121.000	55.667	8.757
3 2 3	46.000	55.667	-1.296
3 3 1	0.000	0.000	-0.000
3 3 2	69.000	69.000	0.000
3 3 3	7.000	7.000	0.000

**Table 5:** Three-group CFA for the comparison of three respondents in their Beer Consumption and Stress-related responses; all comparison triplets are considered

*B S G*	*m*	$\hat{m}$	*z*
1 1 1	263.000	112.667	14.163
1 1 2	3.000	112.667	-10.332
1 1 3	72.000	112.667	-3.831
1 2 1	408.000	229.667	11.767
1 2 2	124.000	229.667	-6.973
1 2 3	157.000	229.667	-4.795
1 3 1	19.000	46.333	-4.016
1 3 2	117.000	46.333	10.382
1 3 3	3.000	46.333	-6.366
2 1 1	21.000	44.667	-3.541
2 1 2	4.000	44.667	-6.085
2 1 3	109.000	44.667	9.626
2 2 1	23.000	87.333	-6.884
2 2 2	182.000	87.333	10.130
2 2 3	57.000	87.333	-3.246
2 3 1	0.000	43.333	-6.583
2 3 2	123.000	43.333	12.102
2 3 3	7.000	43.333	-5.519
3 1 1	1.000	95.000	-9.644
3 1 2	7.000	95.000	-9.029
3 1 3	277.000	95.000	18.673
3 2 1	0.000	55.667	-7.461
3 2 2	121.000	55.667	8.757
3 2 3	46.000	55.667	-1.296
3 3 1	0.000	25.333	-5.033
3 3 2	69.000	25.333	8.676
3 3 3	7.000	25.333	-3.642

Table 2 displays all elements that can be used in the present analysis. Only the constant vector is not shown. It is implied.

The 3 × 3 × 2 cross-classification to be analyzed contains 18 cells. Table 2 contains 18 columns. Together with the constant vector, there would be 19 parameters to be estimated. That is, there are redundancies, and only a selection of the columns in the matrix needs to be included in the base model. The columns in Table 2 include the five main effect vectors (*B*1, *B*2, *S*1, *S*2, and *G*) and the four interaction vectors (*B*1 × *S*1, *B*1 × *S*2, *B*1 × *S*1, and *B*2 × *S*2). The constant vector is implied. The table also contains six vectors that set equal corresponding configurations (see the column vectors 12, 13, 21, 23, 31, and 32 in Table 2, above). The last three vectors blank out the diagonal cells (column vectors 11, 22, and 33). Of these 18 vectors, only the following are used:

1. the four main effect vectors for Beer consumption and Stress;
2. the four interaction vectors Beer consumption × Stress; and
3. the three vectors that blank out the diagonal cells (this decision was discussed above).

In sum, 12 vectors are sufficient to estimate the two-group CFA base model. It has, thus, 6 degrees of freedom and can be statistically evaluated. Table 3 displays the results that are of importance for CFA. For the CFA part of the analysis, we use the *z*-test, and *α* is protected using the procedure proposed by Holland and Di Ponzio Copenhaver (1987), based on the number of corresponding configurations included in an analysis. There is one test for each pair of corresponding configurations. In the present example, we include six corresponding configurations (not blanking out the diagonal cells would result in three additional pairs of corresponding configurations). The nominal *α* is, therefore, protected with respect to six tests.

**Table 3:** Two-group CFA, performed with the new base model

*B S G*	*observed*	*estimated*	*z*
1 1 1	263.000	262.984	0.001
1 1 2	3.000	3.016	-0.009
1 2 1	408.000	266.000	8.707
1 2 2	124.000	266.000	-8.707
1 3 1	19.000	64.000	-5.625
1 3 2	109.000	64.000	5.625
2 1 1	21.000	12.500	2.404
2 1 2	4.000	12.500	-2.404
2 2 1	23.000	23.000	0.000
2 2 2	182.000	182.000	0.000
2 3 1	0.000	61.500	-7.842
2 3 2	123.000	61.500	7.842
3 1 1	1.000	4.000	-1.500
3 1 2	7.000	4.000	1.500
3 2 1	0.000	60.500	-7.778
3 2 2	121.000	60.500	7.778
3 3 1	0.000	0.000	-0.000
3 3 2	69.000	69.000	0.000

The base model used to estimate the expected cell frequencies for the two-group CFA is rejected (LR-*χ*^2^ = 585.70; *df* = 6; *p* < 0.001). We, therefore, can expect discrimination types to emerge. In Table 3, we first note that the frequencies in the diagonal cells (1 1, 2 2, and 3 3) are estimated to equal the observed frequencies. This was intended. When comparing the observed with the expected cell frequencies in the off-diagonal cells, we note that the corresponding cells are estimated to contain the same numbers of responses. The *z*-scores for corresponding cells are, therefore, the same as well.

The most extreme discrimination type is, as before, constituted by Configuration 1 1. The rank order of the *z*-scores of the remaining configurations is not the same as in Table 1, but Configuration 3 1 remains the only one that does not constitute a discrimination type. Overall, the *z*-scores in Table 3 tend to be smaller than in Table 1. Whether or not this reflects a general difference in power will be determined in a power analysis, in subsequent work. Now, we move on to discuss multigroup CFA.

## A Base Model for Multigroup CFA

To derive base models for multigroup CFA, we extend the model used for two-group CFA. The base model for this situation is analogous to the one for two-group CFA. It can include

1. the model constant and all main effects of the cT discrimination variables, that is, 1 + *c*1 - 1 + *c*2 - 1 + … + *c_t_* - 1 column vectors;
2. all interactions among the *c_T_* discrimination variables, that is, (*c*_1_ - 1) ∙ (*c*_2_ - 1) + (*c*_1_ - 1) ∙ (*c*_3_ - 1) +…+ (*c*_1_ -1) ∙ (*c*_2_ - 1) ∙…∙ (*c_T_* - 1) column vectors;
3. a non-redundant selection of vectors that sets equal *G* corresponding configurations (for *G* groups, there are *G* corresponding configurations in each comparison); the total number of possible comparisons is given by the number of cells in a group, *c_T_* = *c*_1_ × *c*_2_ × … × *c_t_* minus the number of cells to be blanked out;
4. the vectors that blank out diagonal cells; when the discrimination variables all have the same number of categories, the number of these vectors is equal to the number of categories; when the discrimination variables differ in their numbers of categories, the number of cells to be blanked out equals the smallest number of categories.

As in the first example of the new approach to 2-group CFA, the decision to blank out diagonal cells does not reflect a characteristic of the new model. Again, this decision was made in the following data example solely to illustrate that the axial symmetry model discussed by Levendosky and colleagues (2011) for repeated observations can also be extended to three and more observations.

As in all applications of CFA in which multiple tests are performed, the significance threshold *α* must be protected based on the number of tests in an analysis. In multigroup CFA, this number is no longer the number of comparisons. Instead, it is the number of comparison groups multiplied by the number of configurations. In confirmatory multigroup CFA, the number of tests is smaller.

## Data Example

In the following data example, we resume the comparison of self-diagnosed alcoholics in the study by Perrine and colleagues (1995). In the first two examples, we compared Respondents 3000 and 3004. Here, we also include Respondent 3007. He also provided answers on 750 consecutive days. We use the same variables as before. The data matrix has 3 × 3 × 3 = 27 cells.

The design matrix for the base model for this example contains, maximally:

1. two vectors each for the main effects of Beer Consumption and Stress;
2. four vectors for the interaction between Beer Consumption and Stress;
3. two vectors for the main effect of Group (the three respondents);
4. six vectors that set equal the cells in each of the triplets of corresponding cells; and
5. three vectors that blank out the diagonal cells 1 1 1, 2 2 2, and 3 3 3 (the reason for this was explained above).

The constant vector is, as before, implied. In sum, these are 18 vectors. However, as in the first example, there are redundancies, and not every one of these vectors is needed for model estimation. Here, we do not need the main effect of the Group variable. When these vectors are not included in the base model, the frequencies are set equal across the categories of the grouping variable. Therefore, we need none of the covariates that set the triplets of cells equal. To fix the diagonal cells, we need five vectors. The model thus has 13 degrees of freedom. For the cell-wise tests, we use the *z*-test. *α* is protected using the Holland and Di Ponzio Copenhaver procedure (1987). For each configuration in a triplet, a test is performed. The number of cell-wise tests is, therefore, in this example, 18. Table 4 displays the results that are of interest in this three-group CFA.

The base model used to estimate the expected cell frequencies for the three-group CFA is rejected (LR-*χ*^2^ = 1550.91; *df* = 13; *p* < 0.001). We, therefore, expect discrimination types to emerge.

Table 4 shows that, as intended, the expected frequencies in the diagonal cells are equal to the observed frequencies. The expected frequencies for the triplets of corresponding cells are equal to each other, also as intended. Now, in contrast to Lienert’s original two-group CFA and also in contrast to the log-linear modeling-based two-group CFA that was introduced in the last section, in three-group CFA cell-wise tests must be performed for each of the cells in a triplet. The reason for this is that it is very well possible that, when three cells are set equal, two of these deviate significantly from average but the third does not. Examples of this can be seen in the triplet of configurations 2 3 1, 2 3 2, and 2 3 3, and in the triplet 3 2 1, 3 2 2, 3 2 3. In the second of these triplets, the first two configurations deviate significantly, with opposite signs, but the third does not. Therefore, it can be said that Configuration 3 2 1 constitutes a discrimination antitype. This indicates that Respondent 3000 provided fewer responses than expected under the null hypothesis that responses with the pattern 3 2 1 are given at equal rates by the three respondents. Configuration 3 2 2 can be said to constitute a discrimination type. It indicates that Respondent 3004 provided more responses than the other two respondents. Configuration 3 2 3 does not deviate significantly. The results for the remaining triplets can be interpreted accordingly.

## Discussion

In this article, we propose a new approach to two- and multigroup CFA. This approach requires the specification of a base model that incorporates the cell-wise group comparisons. The approach comes with a number of important advantages. The first involves that covariates can be taken into account. In the original approach, 2 × 2 tables are set up from the cross-classification of the observed cell frequencies. In the analysis of the resulting tables, there is no room for covariates. In the approach presented here, each of the base models has sufficient degrees of freedom for one or more covariates to be included in an analysis.

A second advantage is that the new approach can straightforwardly be extended to larger numbers of groups. It can also be applied to the comparison of individuals – as illustrated in the present examples -, that is, in person-oriented research, as well as to groups of individuals. In the latter case, the new approach can be used to test hypotheses of group homogeneity which is a key element of person-oriented research concerning groups of individuals (cf. von Eye & Wiedermann, 2024 a).

A third advantage results from the redundancy of certain base models. An important characteristic of CFA base models is that they include all and only the effects that are not of interest to the researcher (Schuster, & von Eye, 2000). As was illustrated above, the redundancy in model specification allows the researcher to select from a number of effects to be included, without change in model fit or expected model frequencies. This implies that researchers can also estimate different model parameters as is needed in functional CFA (von Eye, & Mair, 2008).

A fourth advantage over the original approach to two-group CFA is that the new approach comes with more statistical power. Consider, for example, the three-group CFA example in Table 4. In this example, six triplets of corresponding cells were examined. For each of these, three cell-wise tests were performed. The significance threshold α needed, therefore, be protected with respect to 18 tests in addition to the overall goodness-of-fit test of the base model. Performing three two-group CFAs instead would have required 18 tests as well, but there are three base models, that is, two additional significance tests. As was suggested above, the new approach can result in less extreme protected significance thresholds than the original approach.

When the new approach is applied, it is easy to select cell pairs, triplets or larger groups for comparison. In the examples given above, the diagonal cells were not included in the configural analyses. When, however, researchers are interested in comparing these as well, they can be included. Consider the example in Table 4. In this example, none of the diagonal cells is subjected to tests of the null hypothesis that they are equal. If these tests are of interest, the results given in Table 5 are obtained. The base model used for Table 5 contains only the main effects and all interactions of the discrimination variables. Group-specific variation is not considered. This corresponds to the null hypothesis that the frequencies in the three groups are equal.

This model is most parsimonious. There is no need to include the main effect of the grouping variable anymore, none of the vectors is needed that fix the diagonal cells, and none of the vectors is needed that set equal configurations in triplets. Model fit is poor (LR-*χ*^2^ = 1990.65; *df* = 18; *p* < 0.001), but there are nine additional degrees of freedom. These allow one to test additional hypotheses concerning covariates, as was emphasized above. The number of tests within triplets increases from six to nine. This would also be the number of tests when the original approach is applied three times. The number of follow-up tests of individual configuration increases to 27. This would also be the number of tests performed in standard CFA, under different base models.

The approach proposed in this article can be extended in multiple ways. Here, we discuss three options. First, as was discussed repeatedly in this article, covariates can be considered. This can be achieved by including vectors for continuous covariates in the design matrix, or by extending the cross-classification of analysis so that categorical covariates increase the number of comparison groups.

A second possibility of extending the present approach is to apply it to repeated observations. It can thus be used to answer the question whether there are changes in the same individuals over time. This applies accordingly when changes across location or social contexts are of interest.

The third option to be discussed here concerns the order of CFA (cf. von Eye & Wiedermann, 2024 b). In all approaches to group comparison CFA, thus far, base models posit independence between the discrimination and the grouping variables. Discrimination types or antitypes can, therefore, emerge based on any level of interaction between these variables. It is, however, conceivable that hypotheses are entertained according to which groups differ only in second or higher order interactions. Consider, for example, Leuner’s (1962; cf. Lienert, 1968) study on experimental psychoses. In this study, it was found that no first order but only second order relations exist among narrowed consciousness (*C*), thought disturbances (*T*), or affect disturbances (*A*) that were observed as responses to LSD. When groups are compared in these responses, it could be considered to exclude from the base model the first-order interactions between the response and the grouping variables, that is, the interactions *C* × *G, T* × *G*, and *A* × *G*.

## Data Availability

The data in the tables are free to be used for scholarly purposes.

## Action editor

Lars-Gunnar Lundh served as action editor for this article.

## Open access

This article is distributed under the terms of the Creative Commons Attribution 4.0 International License (http://creativecommons.org/licenses/by/4.0/), which permits unrestricted use, distribution, and reproduction in any medium, provided you give appropriate credit to the original author(s) and the source, provide a link to the Creative Commons license, and indicate if changes were made.
